# Supplementary material for: Encoding of pretrained large language models mirrors the genetic architectures of human psychological traits
Source: medRxiv. 2025 Mar 27:2025.03.27.25324744. Preprint. [Version 1] doi: 10.1101/2025.03.27.25324744 (PMC11974973; doi:10.1101/2025.03.27.25324744)
Supplement: 1 [file NIHPP2025.03.27.25324744V1-supplement-1.pdf]

medRxiv preprint doi: https://doi.org/10.1101/2025.03.27.25324744; this version posted March 27, 2025. The copyright holder for this preprint (which was not certified by peer review) is the author/funder, who has granted medRxiv a license to display the preprint in perpetuity.
It is made available under a CC-BY 4.0 International license .

Supplementary Table 1.

Model Families	Name	Context window size	Embedding dimension	MTEB[*] average score
Open-source[‡]	BERT	512	base: 768<br>large: 1024	BERT$_{base}$: 38.33<br>other: NA
	RoBERTa			NA
Cohere[§]	v2 light	512	1024	NA
	v2 english		4096	NA
	v3.0 light		384	62.0
	v3.0 large		1024	64.5
Google[†]	Gemini	2048	768	66.3[†]
OpenAI[□]	ada v2	8191	1536	61.0
	3-small		1536	62.3
	3-large		3072	64.6

[*] Massive Text Embedding Benchmark (MTEB): a massive benchmark for measuring the performance of text embedding models on diverse embedding tasks, including 56 datasets across 8 tasks [7].

[‡] Scores for BERT$_{large}$, RoBERTa$_{base}$ or RoBERTa$_{large}$ are not published on the MTEB Leaderboard [18].

[§] MTEB scores for embed-english-light-v2.0 and embed-english-v2.0 are not released by Cohere.

[†] The specific MTEB score for Google's text-embedding-004 model is not publicly available. However, the Gecko embedding models [19], which are also part of Google's text embedding family, have reported MTEB average scores of 64.4 for the gecko-1b-256 model and 66.3 for the gecko-1b-768 model.

medRxiv preprint doi: https://doi.org/10.1101/2025.03.27.25324744; this version posted March 27, 2025. The copyright holder for this preprint (which was not certified by peer review) is the author/funder, who has granted medRxiv a license to display the preprint in perpetuity.
It is made available under a CC-BY 4.0 International license .

- Text-embedding-ada-002 and text-embedding-3-small/large are released in 2022 and 2024 separately [20,21].

medRxiv preprint doi: https://doi.org/10.1101/2025.03.27.25324744; this version posted March 27, 2025. The copyright holder for this preprint (which was not certified by peer review) is the author/funder, who has granted medRxiv a license to display the preprint in perpetuity.
It is made available under a CC-BY 4.0 International license .

Supplementary Table 2.

Name	Question items or diagnostic criteria
Neuroticism	Does your mood often go up and down?<br>Do you ever feel 'just miserable' for no reason?<br>Are you an irritable person?<br>Are your feelings easily hurt?<br>Do you often feel 'fed-up'?<br>Would you call yourself a nervous person?<br>Are you a worrier?<br>Would you call yourself tense or 'highly strung'?<br>Do you worry too long after an embarrassing experience?<br>Do you suffer from 'nerves'?<br>Do you often feel lonely?<br>Are you often troubled by feelings of guilt?
Major depressive episode	Depressed mood most of the day, nearly every day, as indicated by either subjective report (eg, feels sad, empty, hopeless) or observations made by others (eg, appears tearful).<br>Markedly diminished interest or pleasure in all, or almost all, activities most of the day, nearly every day (as indicated by either subjective account or observation).<br>Significant weight loss when not dieting or weight gain (eg, a change of more than 5% of body weight in a month), or decrease or increase in appetite nearly every day.

medRxiv preprint doi: https://doi.org/10.1101/2025.03.27.25324744; this version posted March 27, 2025. The copyright holder for this preprint (which was not certified by peer review) is the author/funder, who has granted medRxiv a license to display the preprint in perpetuity.
It is made available under a CC-BY 4.0 International license .

	Insomnia or hypersomnia nearly every day.<br>Psychomotor agitation or retardation nearly every day (observable by others, not merely subjective feelings of restlessness or being slowed down).<br>Fatigue or loss of energy nearly every day.<br>Feelings of worthlessness or excessive or inappropriate guilt (which may be delusional) nearly every day (not merely self-reproach or guilt about being sick).<br>Diminished ability to think or concentrate, or indecisiveness, nearly every day (either by their subjective account or as observed by others).<br>Recurrent thoughts of death (not just fear of dying), recurrent suicidal ideation without a specific plan, or a suicide attempt or a specific plan for committing suicide.
Schizophrenia	Delusions<br>Hallucinations<br>Disorganized speech (e.g., frequent derailment or incoherence)<br>Grossly disorganized or catatonic behavior<br>Negative symptoms (i.e., diminished emotional expression or avolition)
Anorexia nervosa	Restriction of energy intake relative to requirements, leading to a significant low body weight in the context of the age, sex, developmental trajectory, and physical health (less than minimally

medRxiv preprint doi: https://doi.org/10.1101/2025.03.27.25324744; this version posted March 27, 2025. The copyright holder for this preprint (which was not certified by peer review) is the author/funder, who has granted medRxiv a license to display the preprint in perpetuity.
It is made available under a CC-BY 4.0 International license .

	normal/expected).
Autism	Deficits in social-emotional reciprocity, ranging, for example, from abnormal social approach and failure of normal back-and-forth conversation; to reduced sharing of interests, emotions, or affect; to failure to initiate or respond to social interactions.<br>Deficits in nonverbal communicative behaviors used for social interaction, ranging, for example, from poorly integrated verbal and nonverbal communication; to abnormalities in eye contact and body language or deficits in understanding and use of gestures; to a total lack of facial expressions and nonverbal communication.<br>Deficits in developing, maintaining, and understanding relationships, ranging, for example, from difficulties adjusting behavior to suit various social contexts; to difficulties in sharing imaginative play or in making friends; to absence of interest in peers.
Bipolar disorder	A distinct period of abnormally and persistently elevated, expansive, or irritable mood and abnormally and persistently goal-directed behavior or energy, lasting at least 1 week and present most of the day, nearly every day (or any duration if hospitalization is necessary).
Attention-deficit/hyperactivity disorder	Often fails to give close attention to details or makes careless mistakes in schoolwork, at work, or during other activities (e.g., overlooks or misses details, work is inaccurate).<br>Often has difficulty sustaining attention in tasks or play activities

medRxiv preprint doi: https://doi.org/10.1101/2025.03.27.25324744; this version posted March 27, 2025. The copyright holder for this preprint (which was not certified by peer review) is the author/funder, who has granted medRxiv a license to display the preprint in perpetuity.
It is made available under a CC-BY 4.0 International license .

	(e.g., has difficulty remaining focused during lectures, conversations, or lengthy reading).<br>Often does not seem to listen when spoken to directly (e.g., mind seems elsewhere, even in the absence of any obvious distraction).<br>Often does not follow through on instructions and fails to finish schoolwork, chores, or duties in the workplace (e.g., starts tasks but quickly loses focus and is easily sidetracked).<br>Often has difficulty organizing tasks and activities (e.g., difficulty managing sequential tasks; difficulty keeping materials and belongings in order; messy, disorganized work; has poor time management; fails to meet deadlines).<br>Often avoids, dislikes, or is reluctant to engage in tasks that require sustained mental effort (e.g., schoolwork or homework; for older adolescents and adults, preparing reports, completing forms, reviewing lengthy papers).<br>Often loses things necessary for tasks or activities (e.g., school materials, pencils, books, tools, wallets, keys, paperwork, eyeglasses, mobile telephones).<br>Is often easily distracted by extraneous stimuli (for older adolescents and adults, may include unrelated thoughts).<br>Is often forgetful in daily activities (e.g., doing chores, running errands; for older adolescents and adults, returning calls, paying bills, keeping appointments).

medRxiv preprint doi: https://doi.org/10.1101/2025.03.27.25324744; this version posted March 27, 2025. The copyright holder for this preprint (which was not certified by peer review) is the author/funder, who has granted medRxiv a license to display the preprint in perpetuity.
It is made available under a CC-BY 4.0 International license .

	Often fidgets with or taps hands or feet or squirms in seat.<br>Often leaves seat in situations when remaining seated is expected (e.g., leaves his or her place in the classroom, in the office or other workplace, or in other situations that require remaining in place).<br>Often runs about or climbs in situations where it is inappropriate. (Note: In adolescents or adults, may be limited to feeling restless).<br>Often unable to play or take part in leisure activities quietly.<br>Is often 'on the go' acting as if 'driven by a motor' (e.g., is unable to be or uncomfortable being still for extended time, as in restaurants, meetings; may be experienced by others as being restless or difficult to keep up with).<br>Often talks excessively.<br>Often blurts out an answer before a question has been completed (e.g., completes people's sentences; cannot wait for turn in conversation).<br>Often has trouble waiting his/her turn (e.g., while waiting in line).<br>Often interrupts or intrudes on others (e.g., butts into conversations, games, or activities; may start using other people's things without asking or receiving permission; for adolescents and adults, may intrude into or take over what others are doing).
